# Retrospective review to determine the utility of follow-up skeletal surveys in child abuse evaluations when the initial skeletal survey is normal

**DOI:** 10.1186/1756-0500-4-354

**Published:** 2011-09-12

**Authors:** Berkeley L Bennett, Michael S Chua, Marguerite Care, Andrea Kachelmeyer, Melinda Mahabee-Gittens

**Affiliations:** 1 Division of Emergency Medicine, Cincinnati Children's Hospital Medical Center, Cincinnati, Ohio USA; 2 Department of Radiology, Cincinnati Children's Hospital Medical Center, Cincinnati, Ohio USA

**Keywords:** Skeletal survey, Non-accidental trauma, Child Abuse, Follow-up

## Abstract

**Objective:**

The AAP recommends that a follow-up skeletal survey be obtained for all children < 24 months of age who are strongly suspected to be victims of abuse. The objective of the current study was to evaluate the utility of a follow-up skeletal survey in suspected child physical abuse evaluations when the initial skeletal survey is normal.

**Methods:**

A retrospective review of radiology records from September 1, 1998 - January 31, 2007 was conducted. Suspected victims of child abuse who were < 24 months of age and received initial and follow-up skeletal surveys within 56 days were enrolled in the study. Children with a negative initial skeletal survey were included for further analysis.

**Results:**

Forty-seven children had a negative initial skeletal survey and were included for analysis. The mean age was 6.9 months (SD 5.7); the mean number of days between skeletal surveys was 18.7 (SD 10.1)

Four children (8.5%) had signs of healing bone trauma on a follow-up skeletal survey. Three of these children (75%) had healing rib fractures and one child had a healing proximal humerus fracture. The findings on the follow-up skeletal survey yielded forensically important information in all 4 cases and strengthened the diagnosis of non-accidental trauma.

**Conclusion:**

8.5 percent of children with negative initial skeletal surveys had forensically important findings on follow-up skeletal survey that increased the certainty of the diagnosis of non-accidental trauma. A follow-up skeletal survey can be useful even when the initial skeletal survey is negative.

## Background

Skeletal surveys are an important part of the evaluation of young children who are suspected to be victims of non-accidental trauma and are a major part of the diagnosis of physical abuse [1]. The American Academy of Pediatrics Committee on Child Abuse and Neglect recommends that a skeletal survey be obtained for any child less than 24 months of age with suspected physical abuse [2]. This recommendation is supported by The American College of Radiology [3] and skeletal surveys are considered optimal for the initial evaluation of suspected victims of physical abuse. A follow-up skeletal survey approximately 2 weeks after the initial presentation has been recommended to detect injuries that were not visible on the initial radiographs, differentiate suspected fractures from normal variants, and possibly provide a timeframe in which injuries occurred [2-5].

In three separate studies, Kleinman et al [4], Zimmerman et al [6] and Harlan et al [7] showed that additional radiographs identified new fractures and clarified tentative findings in children with abnormalities in the initial skeletal survey. These investigations demonstrated the utility of obtaining follow-up imaging in children with abnormal initial skeletal surveys. The data from Zimmerman et al showed that all children who had a normal initial skeletal survey also had a normal follow-up skeletal survey [6]. In contrast, the study by Harlan et al revealed that 12% of their patients with a normal initial skeletal survey had abnormalities on the follow-up skeletal survey [7]. Our objective was to address this discrepancy regarding the utility of a follow-up skeletal survey in this population. Because a follow-up skeletal survey induces exposure to radiation, further information regarding the yield of this testing can be valuable for medical practitioners.

## Methods

Cincinnati Children's Hospital Medical Center (CCHMC) Institutional Review Board approval was obtained prior to study commencement and a waiver of consent was granted. A retrospective review of radiology records from September 1, 1998 - January 31, 2007 at CCHMC was performed. Skeletal survey radiographs were obtained according to American College of Radiology, Standards for Skeletal Surveys [8] and consisted of 19 images including anteroposterior and lateral views of the axial skeleton and tightly collimated views of the appendicular skeleton. Oblique views of the ribs were not routinely performed. Digital radiographs were reviewed on a picture archiving and communication system (PACS) workstation and interpreted by board certified pediatric radiologists at the time of diagnosis.

### Inclusion Criteria

A child was included in the study if he or she was < 24 months of age, suspected to be a victim of child physical abuse as recorded in the medical record, and received an initial and follow up skeletal survey within 56 days. This time frame was based on previous literature [6] and CCHMC radiologist recommendations recognizing that many fractures may have completely healed after 56 days and at that time would not be detectable on radiographs. Children whose initial skeletal survey had no signs of bone dysplasia and no indications of skeletal trauma were included for further analysis. Skeletal surveys were considered the definitive study for skull fractures; patients with computed tomography (CT) findings that suggested a skull fracture but who had a negative initial skeletal survey were still considered eligible. Patients with diastasis of sutures without other evidence of skeletal trauma were also considered to have findings consistent with a negative study; thus they were also eligible for study inclusion.

### Exclusion Criteria

Patients were excluded if their skeletal survey was obtained for reasons other than concerns for abuse (such as bone dysplasia or metabolic disease) or if the results of the skeletal survey were unavailable. An initial skeletal survey was considered positive and therefore excluded from further analysis if a fracture was identified or if findings suspicious for fracture defined as non-physiologic periosteal reaction or bony irregularity that necessitated a follow-up study. Patients who only had an initial skeletal survey without a follow-up skeletal survey were excluded. Based on previous literature,[6] and CCHMC radiologist recommendations, patients with follow-up skeletal surveys completed greater than 56 days after the initial skeletal survey were also excluded. This cut-off was made recognizing that some fractures may have completely healed during that time frame making the comparison of the two sets of radiographs inappropriate for the purposes of this study.

## Results

During the 101 month study period, 47 children had a negative initial skeletal survey and a repeat skeletal survey within a 56 day period and were therefore included for analysis. Details regarding the timing of the follow-up skeletal survey are included in Table 1.

**Table 1:** Timing of follow-up skeletal survey for children with a negative initial skeletal survey who had a follow-up skeletal survey within 56 days

Timing of Follow-up Skeletal Surveys	
**Number of Patients (%)**	**Timing of Follow-up**

14 (29.7%)	9-13 days

21 (44.6%)	14-21 days

8 (17.2%)	22-40 days

4 (8.5%)	40-56 days

The mean age in this population was 6.9 months (SD 5.7), and there were 30 (64%) males and 17 (36%) females. The median number of days between skeletal surveys was 18.7 (SD 10.1).

Forty-three patients had a negative initial skeletal survey and a negative follow-up skeletal survey. The skeletal surveys were obtained because of concerns for abuse, either by history, physical exam, or imaging findings. Eleven of these children (26%) presented with cutaneous bruising and no other findings. Thirty children (70%) had an acute intracranial injury without evidence of fracture. The other 2 patients had concerns for abuse by history.

Four children (8.5%) had evidence of bony injury on the follow-up skeletal survey. Three children (75%) had healing rib fractures and one child had a healing proximal humerus fracture. The follow-up skeletal surveys yielded forensically important information in all 4 patients.

The number of positive follow-up skeletal surveys was analyzed to determine the predictive value of a completely negative skeletal survey in this specific population in which the multi-disciplinary team was concerned about abuse. A negative initial skeletal survey had a true negative rate of 91.5% (95% CI 80-98) and a false negative rate of 8.5% (95% CI 2.4-20.4).

## Discussion

In this study, four (8.5%) of the children with negative initial skeletal surveys had forensically important findings on the follow-up films and 1 patient had a fracture requiring clinical management. In all of these cases, the information gained by the follow-up skeletal survey strengthened the diagnosis of non-accidental trauma which supported the subsequent safety plans for these children. Our findings and other research supports and affirms the AAP recommendations that all children < 24 months of age receive follow-up imaging when abuse is strongly suspected [2]. In addition, the results of this study suggest that in cases when abuse is strongly suspected, follow-up imaging should be pursued regardless of the results of the initial evaluation.

The ALARA principle states that every reasonable effort should be made to maintain exposures to ionizing radiation as far below the dose limits as practical. This is an important consideration for follow-up imaging. In this study, 75% of the patients who had findings on follow-up skeletal survey had rib fractures. The frequent identification of occult rib fractures on follow-up imaging has been noted in other studies. Zimmerman et al noted that 66% of fractures detected on follow-up skeletal surveys were rib fractures [6]. Kleinman et al noted that 94% of additional injuries noted on follow-up skeletal survey were rib fractures or classic metaphyseal lesions [4]. Similarly, Harlan and colleagues found that 49.7% of fractures found on follow-up skeletal survey were rib fractures [7]. The data from our study and previous investigations demonstrate that chest radiographs have a high yield for detecting occult injuries on follow-up skeletal surveys. The aforementioned study by Harlan determined that a limited follow-up skeletal survey could be performed without missing clinically significant new fractures [7]. When limiting radiation is a priority, a chest radiograph in lieu of a full skeletal survey may be useful. Future studies are needed to further explore the optimal number of images in follow-up skeletal surveys.

A larger sample size may have produced a narrower confidence interval and increased the reliability of our results. Future studies using multiple centers are considered to address this further.

The literature states that a repeat skeletal survey should be completed approximately 2 weeks after the initial skeletal survey [3,4], but variations in the timing of follow-up are to be expected. The follow-up skeletal survey was completed between 9-13 days in 14 (30%) of our patients and after 40 days in 4 (8.5%) of our patients. Two of these 18 children had positive findings on the follow-up evaluation. It is possible that some fractures may not have been detected with early follow-up or healed and subsequently missed with delayed follow-up. Therefore our results may underestimate the frequency of findings on follow-up skeletal surveys in this population.

The skeletal surveys were read by the attending pediatric radiology staff at our institution and therefore slight variability between radiologist interpretations may exist. While utilizing a single radiologist for all skeletal surveys would limit this potential variability, our methods reflect clinical practice and allow the results to be generalized to a larger population.

## Conclusions

Forty-seven children with an initially negative skeletal survey were included for analysis of the utility of the follow-up skeletal survey. Four patients (8.5%) had forensically important findings on the repeat skeletal survey including healing rib fractures in 3 patients (75%). This study adds to the literature by further addressing the yield of follow-up imaging in patients with suspected non-accidental trauma. A follow-up skeletal survey can be valuable for children who are suspected victims of abuse even when the initial skeletal survey is negative.

## Competing interests

The authors declare that they have no competing interests.

## Authors' contributions

BB conceptualized and designed the study, collected and analyzed the data and prepared the manuscript. MSC designed the study, collected the data and assisted with manuscript preparation and review. MC assisted with study design, reviewed the radiology results, assisted with manuscript preparation and review. AK assisted with data collection and reviewed the manuscript. MMG assisted with analysis, manuscript preparation and review. All authors read and approved the final manuscript

## References

[B1] Kemp AM Which radiological investigations should be performed to identify fractures in suspected child abuse? Clinical Radiology 2006 61 723 736 10.1016/j.crad.2006.03.017 16905379

[B2] Sane SM American Academy of Pediatrics, Section on Radiology: Diagnostic Imaging of Child Abuse Pediatrics 2000 105 1345 134810835079

[B3] Di Pietro MA AAoPSo R Diagnostic Imaging of Child Abuse Pediatrics 2009 123 1430 1435 19403511 10.1542/peds.2009-0558

[B4] Kleinman PK Follow-up skeletal surveys in suspected child abuse AJR Am J Roentgenol 1996 167 893 896 8819377 10.2214/ajr.167.4.8819377

[B5] Kleinman PK Kleinman PK Skeletal Imaging Strategies Diagnostic Imaging of Child Abuse 1998 2 Mosby Incorporated: St. Louis 237 241

[B6] Zimmerman S Utility of follow-up skeletal surveys in suspected child physical abuse evaluations Child Abuse and Neglect 2005 29 1075 1083 10.1016/j.chiabu.2004.08.012 16315349

[B7] Harlan SR Follow-up skeletal surveys for nonaccidental trauma: can a more limited survey be performed Pediatr Radiol 2009 39 962 968 10.1007/s00247-009-1313-7 19565233

[B8] Applegate KE ACR practice guideline for skeletal surveys in children American College of Radiology 2006 203 207

